# Anterior abdominal wall reconstruction with mesh implants: indications and limitations in a developing tropical economy

**DOI:** 10.11604/pamj.2020.37.57.25107

**Published:** 2020-09-15

**Authors:** Aloysius Ugwu-Olisa Ogbuanya, Ugochukwu Uzodimma Nnadozie, Livinus Nnanyerugo Onah, Stanley Nnamdi Chinedu Anyanwu, Anastasia Amechi Mmeke

**Affiliations:** 1 Department of Surgery, Alex Ekwueme Federal University Teaching Hospital, Abalaliki, Ebonyi State, Nigeria,; 2 Department of Surgery, Ebonyi State University, Abakaliki, Ebonyi State, Nigeria,; 3 Department of Obstetrics and Gynecology, Enugu State University Teaching Hospital, Enugu, Nigeria,; 4 Department of Surgery, Nnamdi Azikiwe University, Awka, Anambra State, Nigeria.

**Keywords:** Abdominal, defect, implant, reconstruction, ventral

## Abstract

**Introduction:**

the role of surgery in managing massive midline abdominal wall defects has continued to rise, leading to higher demand for more effective techniques in order to limit recurrences. There is paucity of data on this subject in Southeast Nigeria. The aim of this study is to document the indications and challenges of treatment of complex, midline abdominal wall defects in our centre.

**Methods:**

this was a cross-sectional study of adult patients with complex, midline abdominal wall defects managed with mesh implants over a five-year period.

**Results:**

a total of 182 adult patients, predominantly females 160(87.9%), received mesh implants for complex abdominal wall defects. The common indications were incisional hernia 128(70.3%), abdominal wound dehiscence 16(8.8%) and divarication of recti 16(8.8%). About one-third 62(34.1%) of the patients required additional abdominoplasty procedure. Delay towards prompt surgical repair was noted in 168(92.3%) patients, notably due to financial constraints 32(17.6%) followed by comorbidities requiring serial assessments 24(13.2%). Superficial wound infection rate was 5.5% while deep (mesh) infection was noted in two (1.1%) patients. Recurrence and perioperative mortality rates were 1.1% and 1.6% respectively. Diabetes mellitus in obese female patients was an independent predictor of perioperative death (p=0.000).

**Conclusion:**

the most common indication for abdominal wall reconstruction in our environment is incisional hernia. The use of prosthetic meshes to repair complex abdominal wall defects is largely safe and effective in our practice, but timely reconstruction is commonly hampered by multi-faceted economic, clinical and pathological barriers.

## Introduction

Anterior abdominal wall defects encompass a heterogeneous array of musculo-fascio-cutaneous abnormalities that may present a wide range of clinical behaviors [1,2]. The defects are commonly produced from infections, herniation, tumor extirpation, trauma and less frequently from congenital abdominal wall disorders [3-5]. The clinical entities encountered under this discourse include incisional hernia (IH), divarication of recti otherwise called ‘diastasis recti abdominis’ (DRA), giant primary midline abdominal wall hernias and defects that accompany abdominal wound dehiscence, tumor resection and open abdomen following damage control surgery [3,6,7]. Increased attention to the clinico-pathologic disturbances, along with changes in the surgical management of these defects have led to an exponential growth in research relating to abdominal wall reconstruction in recent years [1,4,8]. It has been observed that most developing countries are today experiencing an increasing incidence of non-communicable diseases like incisional hernias, cancers (resection may cause large abdominal wall defects), trauma (surgery may lead to open abdomen) and obesity (predisposes to hernia and abdominal wall defects) [9,10]. Many of the abdominal defects resulting from the above pathologic processes are voluminous and published data indicate that when repaired by suture-based method, they often recur with higher clinical and re-operative risks [2,3,10]. The role of surgery in managing these diseases has continued to rise, leading to higher demand for more effective and proper surgical principles and techniques [3,4,9].

The critical issue with these complex abdominal wall defects is the high propensity for recurrence if the repair is suboptimal [1,3,4]. This is particularly relevant in Sub-Saharan Africa with neglected, longstanding, voluminous, ventral hernias and the often-associated ignorance, poverty, deplorable health facilities and dearth of surgical personnel [10,11]. The use of autogenous materials in the reconstruction of such difficult abdominal wall defects has a long history, but the advent of prosthetic implants has diminished their clinical relevance in the developed nations [3-5]. Nevertheless, suture repair has remained popular in repairing these defects in many parts of Africa mainly due to poverty and surgeons´ preferences [10]. The popularity of tension-free repair with prosthetic implants over the traditional anatomic, suture-based methods has been ascribed to its lower recurrence, shorter length of hospital stay (LOHS), superior quality of life and better cosmetic outcomes [2,12,13]. Indeed, there is a global trend towards tensionless repair of abdominal wall defects with meshes, but in many parts of Africa, the legendary suture-based methods are still commonly used despite the large accumulated pools of untreated abdominal wall hernias that have been overlooked and better served with prosthetic implants [10,11,14,15]. Despite an increasing application of meshes to reconstruct complex abdominal defects in our centre in recent years, no organized study has been done to report the experiences so far. Moreover, no original article on the use of mesh for complex abdominal defects other than hernias has been published in Southeast Nigeria. The aim of this study is to document the spectrum and challenges of treatment of complex, midline abdominal wall defects in our centre.

## Methods

This is a cross-sectional analytical study of all consecutive patients that fulfilled the inclusion criteria and were surgically managed for large or complex midline abdominal wall defects from January 2013 to December 2017. The study was done in Alex Ekwueme Federal University Teaching Hospital, Abakaliki (AEFUTHA), South east Nigeria. Initially, all adult patients aged 18 years and above who presented with midline abdominal wall defects were seen and counselled for mesh repair. However, the focus of this current study is on complex midline abdominal wall defects namely large defect sizes, recurrent and multiple defects. Though, some patients with small midline defects received mesh implants during the period of this review, they were not included or further evaluated in this study.

Patients presenting with single large (>4.0cm in widest diameter) incisional or primary midline abdominal wall hernias were included. Also, those with multiple (incisional or primary including epigastric, umbilical or paraumbilical) midline hernias of any sizes were included. All patients with divarication of recti, abdominal wound dehiscence (later converted to ventral hernia through healing by secondary intension or skin grafting) and those with large residual defects after resection of abdominal wall tumors were included. All recurrent midline hernias or defects were also included. Patients with obstructed or strangulated hernias at time of presentation were excluded. Also, those with metastatic abdominal wall or intra-abdominal malignancies and those who failed to give consent were excluded. Patients with American Association of Anaesthesiologists (ASA) score more than ASA II were excluded. Informed consent was obtained from all the patients before recruitment into the study.

All the patients presenting with midline abdominal wall defects were initially interviewed and examined to select those that fulfill the inclusion criteria. Only the selected patients who received mesh implants were further evaluated. However, only 182 of the selected patients accepted and gave consent for mesh implants, the rest declined mesh for reasons ranging from financial impediments to socio-cultural barriers. The 182 patients formed our study population. Each patient was thoroughly interviewed and his or her socio-demographic data and clinical details noted and entered into a proforma. Emphasis was on presenting complaints, duration, and history of previous abdominal trauma, operations or hernia repairs, number of pregnancies, occupation and family history of similar defects. On examination, the type, site, number and size of the defects and state of surrounding skin were recorded.

Evidence of factors perpetrating raised intra-abdominal pressure (bladder outlet obstruction, chronic cough, ascites, intra-abdominal masses, obesity) was sought and noted. Basic investigations and special tests like abdominal ultrasound, chest x-ray, computed tomography and ECG were reserved for older patients and those with comorbidities. Pre-operatively, malnutrition and anaemia were corrected and all active infections treated. Prophylaxis for deep venous thrombosis (DVT) was commenced where applicable and all the patients were counseled for mesh placement using polypropylene mesh (PROLENE mesh, Braun Inc.). Prophylactic antibiotic was given intravenously at induction of anaesthesia. Vertical midline incision (elliptical where appropriate) was used in most cases and dissection developed to the fascial plane using combined diathermy and instrument dissections. The sac, when present, was mobilized and opened. The content was noted, released and returned into the peritoneal cavity. Adhesions were lysed and edges of the recti freed from bands and omentum. Abdominoplasty was added in those with massive truncal adiposity. Monofilament nylon 2 was used to approximate the recti routinely except when not practicable, followed by a reinforcing, large onlay mesh implant anchored with nylon 2/0 suture. Tube drain was routinely inserted and wound closed in layers.

Skin sutures were removed on the 12^th^-14^th^ post-operative day. The patients were actively followed up for 24 months. Follow up visits were arranged initially at two weeks after discharge, followed by one-month interval for three times, then every three months for another three times. Thereafter, patients were given appointments every six months till 24 months from the time of hospital discharge. Telephone interview were arranged for patients who defaulted from follow up in two consecutive periods. A new appointment schedule, commonly the next clinic date was often arranged. During follow up, post-operative evaluation involved search for respiratory insufficiency, seroma, haematoma, wound infections, perioperative deaths and length of hospital stay (LOHS) in the early postoperative period; then, recurrences, hypertrophic scar and tumour implantation in the later part of the follow up period. Overall, 4(2.2%) and another 5(2.7%) patients were lost from both clinic visit and telephone interview at 18 and 24 months of follow up respectively. Data were analyzed using Statistical Package for Social Sciences (SPSS) software version 22.0 (IBM, Chicago, IL USA, 2015) and presented as means, standard deviation, percentages and tables. Chi-square (χ2) test was used to measure some categorical variables. Confidence interval was calculated at 95% level and significance at 5% probability level (p<0.05). Formal approval was obtained from the institutional Ethical Review Board of AEFUTHA before commencement of this study. All ethical principles relating to studies on human subjects were observed during the study period.

## Results

During the period under review, 512 adult patients with midline abdominal wall defects were seen, but only 322(62.9%) patients fulfilled the inclusion criteria. Of the 322 patients, only 182 (56.5%) consented to prosthetic mesh implantation and formed our study population. The ages of the 182 patients ranged between 18-75 years with a mean of 40.19±SD 12.84. The 182 patients were evaluated and subsequently had operative repairs for various types of large and complex midline abdominal wall defects. There were 22 (12.1%) males and 160 (87.9%) females. There was statistically significant difference (p=0.000) with respect to sex of the patients. There were 140 multiparous women. Multiparity in premenopausal women was an independent predictor of development of complex abdominal wall defect (p=0.003). The commonest indication for the reconstructions was incisional hernia (68.1%) while the least was a defect arising from tumor extirpation in a 46year old male patient, which accounts for 0.5% of all reconstructions (Table 1). The vast majority 121(97.6%) of IH were referred from private, mission and general hospitals and majority 116(93.5%) followed caesarean section. In about one-third 62(34.1%) of the patients, abdominoplasty (excess subcutaneous fat excision/panniculectomy, releasing incisions on rectus sheath and excision of redundant skin) was added to the mesh implantation. More than nearly three-quarter 45(72.6%) of the patients who received abdominoplasty were obese. Complex abdominal wall defect in obese female patients is a predictive index of additional abdominoplasty requirement during prosthetic mesh implantation (p=0.020).

**Table 1:** indication for abdominal wall reconstruction

Indication	Male	Female	Total (%)
Incisional hernia	4	124	128 (70.3)
Divarication of recti	2	14	16 (8.8)
Abdominal wall tumor resection	0	1	1 (0.5)
Abdominal wound dehiscence	10	6	16 (8.8)
Giant para/ umbilical hernia	4	11	15 (8.2)
Giant epigastric hernia	2	4	6 (3.3)
Total	22	160	182 (100.0)

The major reasons for requesting surgical corrections by the patients included cosmetics 124(68.1%) and abdominal pain 22(12.1%). Other reasons were fear of future disease related to the defect 6(3.3%), easy satiety 2(1.1%) and a combination of reasons 28(15.4%). The vast majority 168(92.3%) of the patients experienced delay in having their surgeries performed promptly. These delays were multifactorial in about one-fifth 36(19.8%) of the patients, but the single most frequent reason for postponing the operations was financial impediments 32(17.6%), followed by comorbid illnesses that necessitated serial assessments (Table 2). A total of 120 comorbid conditions were recorded in 78 (42.9%) patients. Twenty-four (30.8%) patients harbored multiple (two or more) comorbidities. The comorbidities were obesity (46), hypertension (40), diabetes (16), chronic obstructive pulmonary disease-COPD (11), benign prostatic hyperplasia (2), HIV/AIDS (2) and one each of pulmonary tuberculosis, simple goiter and chronic renal disease. The frequency and types of anaesthesia used for repair were related to types of the defects (Table 3). More than half 110(60.4%) had ASA I score; the rest 72(39.6%) had ASA II. Majority 3(60%) of the conversion from spinal to general anaesthesia were due to long operating time necessitating further pain control and skeletal muscle relaxation.

**Table 2:** reasons for delay before surgery

Reasons for delay before operation	Frequency	Percent (%)
Financial constraint	32	17.6
Delay from comorbidities	24	13.2
Lack of bed space	8	4.4
Default from clinic visit	15	8.2
Anesthetic bureaucracy	20	11.0
Theatre logistics	18	9.9
Industrial disharmony	5	2.7
Laboratory bottlenecks	10	5.5
Multifactorial reasons	36	19.8
Prompt operation	14	7.7
Total	182	100.0

**Table 3:** effects of abdominal wall defect on choice of aneasthesia

Method	Frequency	Incisional	Divarication	Others	Percent (%)
General	147	94	16	37	80.8
Spinal	22	22	0	0	12.1
Spinal+sedation	3	3	0	0	1.6
LA+sedation	1	0	0	1	0.5
Epidural	4	4	0	0	2.2
Spinal (converted to GA)	5	5	0	0	2.8
Total	182	128	16	38	100.0

* LA= local anaesthesia; †GA= general anaesthesia ‡Others=giant epigastric hernia, giant umbilical/paraumbilical hernia,abdominal wound dehiscence,post-tumor resection

The complications of the reconstructions are shown below (Table 4). The perioperative mortality rate recorded in this study was 1.6% (three patients); two deaths were from sepsis in diabetic patients and one from hyperglycemic crisis. Diabetes mellitus in obese patients aged 45 years and above was an independent predictor of perioperative death (p=0.000). A fourth death, due to complications of diabetes mellitus, occurred in the seventh month of follow up. The two (1.1%) recurrences were on one patient with deep mesh infection and another one surgical site infection (apparently non-mesh). There was statistically significant difference (p=0.001) in the rate of recurrence between cases with infection-related multiple wound events and those without wound infection. The LOHS ranged from three days to six weeks. About a tenth 19(10.4%) of the patients stayed beyond two weeks on hospital admission; majority 12(63.2%) were those that received abdominoplasty in addition to mesh implantation, the rest were due to wound infections, tumor implantation and concurrent illnesses.

**Table 4:** post-operative outcomes

Complications	Frequency	Percent (%)
Wound infection	10	5.5
Seroma	13	7.1
Prolonged ileus	14	7.7
Bowel injury	1	0.5
Reactionary haemorrhage	3	1.6
Deep vein thrombosis	1	0.5
Bladder injury	1	0.5
Deep (mesh) infection	2	1.1
Tumor implantation	1	0.5
Recurrence	2	1.1
Total	46	25.3
**Length of hospital stay (days)**		
1-3	80	44.0
4-7	46	25.3
8-10	23	12.6
11-14	14	7.7
>14	19	10.4
Total	182	100.0
**Mortality**	3	1.6

## Discussion

The anterior abdominal wall is a complex, composite structure that poses a challenge to the reconstructive surgeon. Several reconstructive techniques have been described using autologous tissue and prosthetic materials with varying results, availability and cost implications [5]. In this series, our patients comprised predominantly young and middle-aged people with a preponderance of multiparous females that presented electively at the specialist surgery clinic. Put differently, approximately 10 out of every 12 patients we managed were females and 35 out of every 40 women evaluated were multiparous. Overall, IH (70.3%) was the commonest indication for the abdominal wall reconstruction in this study. It has been cited that IH occurs in approximately 5-15% of laparotomies and may rise to 26% in the context of wound sepsis [16-21]. This is followed, simultaneously by divarication of recti and abdominal wound dehiscence, each accounting for less than a tenth (8.8%) of the reconstructions done (Table 2). These findings are similar to previous results from Nigeria, South Korea, Yemen, Europe and Saudi Arabia [1,13,16-18,22-24]. Majority (68.1%) of our patients reported that cosmetic correction is the primary reason they sought reconstruction, followed by abdominal pain (12.1%). This is comparable to results from Saudi Arabia where 63.4% of the patients stated cosmetics as their major reason for seeking repair, followed by abdominal pain (24.1%) [24].

Financial impediment was the single most important reason for the delay accounting for 17.6% of all causes of delay. In Northeast Nigeria, Gali and colleagues observed that 80% of their patients were delayed for at least two years before presentation, principally due to financial constraint, delayed referral and interference by non-medically qualified persons [16]. The recurrence rate of 1.1% recorded in this study is comparable to rates of 2.6% reported by Ezeome and Nwajiobi in Nigeria [3], but higher than 0.0% quoted in Saudi Arabia [24]. Agbakwuru and colleagues reported far higher recurrence rate of 9.1% from Ile-Ife, Nigeria [18]. There are several reasons why incisional hernia is the most common indication for reconstruction of complex abdominal wall defects in our environment. The frequent relationship between incisional hernia and caesarian section or gynecological operations has a historical pedigree, and reasons adduced can be either surgeon or patient (disease) related. Importantly, the lower rank of surgeons and general duty doctors that perform caesarean sections, often in emergency situations, were shown to execute the abdominal wound closure hurriedly, using absorbable sutures to close the fascia [16,18,21]. These practices, together with midline incisions and non-mass closure techniques, among other technical errors, have been implicated as major determinants of incisional hernia development after abdominal operations [16,22]. In this survey, 87.5% of the women were multiparous, which overlapped with the value of 85.6% reported in Middle East [24]. Published clinical data indicate that multiparity and obesity contribute to laxity of abdominal wall and may predispose to both incisional hernia after abdominal operations or divarication of recti [8,12,17].

In our series, the recurrence rate was low (1.1%) compared to 20-46% cited in non-mesh series [16]. This achievement probably was related to pre-operative weight reduction in obese patients, clearance of chest infections and respiratory exercises to prevent cough in the pre- and post-operative periods, judicious use of wound drain, initial mass closure with stout nylon when feasible and prosthetic mesh implantation. It is noteworthy that two cases of mesh infections were encountered, however, recurrence and mesh removal occurred in one of the two, but the second case succumbed to conservative treatment with antibiotics. In Enugu, Nigeria, mesh infection was also responsible for repair failure (recurrence) rate of 2.6% that necessitated re-operation after 38 months [3]. In Ile-Ife [18], Nigeria, the higher recurrence rate of 9.1% may be partly explained by the fact that all the 44 cases were repaired by non-mesh, suture-based methods. Moreover, the higher wound infection rate of 11.4% compared to 5.5% in the current study is clinically significant, because all the patients that developed recurrence in the Ile-Ife [18] series had wound infection. In Turkey and Saudi Arabia however, there were no cases of recurrences after abdominal wall reconstruction during the 24 months period of follow up [12,24]. The availability of laparo-endoscopic services, pre-operative weight reduction, use of mesh implants, uniform reconstruction under general anaesthesia and wearing of abdominal binder (corset) for at least four weeks postoperatively were considered important steps that may have significantly limited the development of recurrence in those reports [12,24].

The impact of delayed presentation on overall outcome after development of midline abdominal wall defects has been highlighted by several workers [3,7,16,18]. Over time, the defects enlarge in size and the contents may acquire accessory vasculature (making repair more difficult and hazardous), become strangulated or eviscerate and occasionally lead to enterocutaneous fistulae [2,8,13,16,18]. In extreme situations where the defects are very voluminous, the intra-abdominal viscera may forfeit their right of domicile, leading to ‘loss of domain’ [3,7]. Postoperatively, many of the patients with ‘loss of domain´ may develop abdominal compartment syndrome (ACS) [3,7]. On a happier note, none of our patients developed severe ACS that necessitated respiratory support, though the reasons are not yet clear. In Enugu, Nigeria, delayed presentation allowed formation of giant hernias, which subsequently led to ACS and postoperative respiratory distress in three patients [3]. Incidentally, two (4.9%) deaths recorded in that series were both due to respiratory distress from ACS [3]. The three perioperative deaths in this survey were all linked to diabetic complications and this may be explained by the high proportion of obesity co-existing with diabetics in this series.

We utilized mesh implants to reconstruct the defects in the patients in this series because they were selected on the basis of adverse markers of high risks of recurrence after repair. These indices included recurrent and re-recurrent hernias, multiplicity of defects, extensive sizes, massive abdominal wall fats, prospects of future pregnancies in previously grand multiparous women and precipitating comorbid conditions (chronic bronchitis, bladder outlet obstructions, abdominal wall neoplasm and chronic renal failure). These observations and repair approach were akin to the experiences of Ezeome and Nwajiobi in Nigeria following examination of 41 patients (28 females, 13 males) selected for the large sizes of their abdominal wall hernias, over a six years period [3]. Although suture-based fascial apposition may be done in smaller defects (<4cm in width), unfortunately, the recurrence rate following such repairs is estimated to be in excess of 50% [2].

In the current review, the numerous tissue-based techniques like Mayo´s technique, Keel´s operation, and component separation technique, use of tensor fascia lata as tissue replacement or vascularized pedicle flap were not employed during the operative repair. Previous investigators have documented drawbacks associated with these techniques [2,5]. For instance, the free or pedicle tensor fascia lata flap creates a donor site with potential morbidities [2,5]. In Enugu, Nigeria, the use of tensor fasciae lata to manage a large ventral hernia led to a repair failure and recurrence under one year, prompting the authors to advise extreme caution with the use of tissue-based approaches for repair of large ventral hernias [3]. Nevertheless, we found the use of mesh easier and faster than any of the tissue-based techniques aforementioned, though we have not dismissed the usefulness of the versatile autogenous tissue replacement option, namely component separation technique of Ramirez [2,14] and its modifications that employ local transposition of rectus muscles. Since its original description by Ramirez and associates in 1990, the technique has been increasingly used as a tensionless closure of extensive, full thickness anterior abdominal wall defect with autologous tissue [1,8,13,14]. In its classic form, the medial edge of external oblique aponeurosis is released, followed by separation of rectus abdominus muscles to achieve rectus muscle advancement ranging from 10-35cm [8,13,14]. Many studies recommend additional application of synthetic mesh in an onlay fashion to supplement the attenuated layers of the anterior abdominal wall after the Ramirez procedure [1,8,13].

In those with wound dehiscence, we allowed healthy granulation tissue to grow over the intra-abdominal viscera and seal off any residual space interfacing between the peritoneal cavity and atmospheric space. A recent update for repair of complicated hernia recommends use of sutures or biological meshes in contaminated classes III and IV surgical wounds, but synthetic meshes for classes I and II wounds [15,25]. However, the duo of prohibitive prices of biological meshes and controversies surrounding use of conventional prosthetic meshes in contaminated fields informed our decision to employ watchful waiting namely conservative management till such a time, the use of prosthetic devices was deemed safe in these cohorts with abdominal wound dehiscence. For the tumor resection, our decision to implant mesh was timely, because the resultant defect at the time of surgery was not amenable to suture-based closure. Experience from Hamilton, USA on 22 patients with complex abdominal wall hernias (CAWH) following cancer surgery was comparable to ours [6]. The patients in our series and US study [6] expressed worries pre-operatively with respect to the impact of the postoperative defects created after major resection of their anterior abdomen. This perhaps allowed the US authors to conclude that CAWH have a substantial impact on the quality of life of cancer patients and that hernia management should form an integral part in the spectrum of cancer treatment [6].

The use of general and spinal aneasthesia was far more frequent than local infiltrative anaesthesia, probably due to a need for adequate relaxation. Traditionally, abdominal operations requiring extensive abdominal wall manipulations require general anaesthetic techniques to produce adequate muscle relaxation and hence patients´ cooperation and surgeon´s satisfaction. Though earlier workers have expressed concern over the inordinate use and propensity for general and spinal anaesthesia for hernia repair by African surgeons, reconstruction of complex abdominal wall defects with mesh under general or regional anaesthesia is not an Africa phenomenon [10,11,18]. However, emerging evidence from published data in Italy indicate that local anaesthesia for mesh repair of incisional hernia is feasible, safe and effective [25]. The researchers noted that over half (71, 55.0%) of the 129 patients with incisional hernias were fixed under local anaesthesia, with only two (2.8%) requiring conversion to general anaesthesia. However, careful analysis showed a caveat, with the authors selecting only elective and reducible hernias, and those with defect diameter less than 40cm [25]. In a nutshell, the authors, through design, minimized sources of complexities and indeed major requirements for general or regional anaesthesia at outset. Much concern and reservation still exist on the applicability of local anaestheia for complex abdominal wall defects as the authors noted that 20 patients (28.2%) needed minor sedation or analgesia and another seven (9.9%) requested for major sedation [25]. This study presents two main strengths. First, to the best of our knowledge, the present study is the pioneer investigation in the Ebonyi province of Southeast Nigeria to document the use of prosthetic implants to reconstruct complex midline abdominal wall defects. Second, this study comprised the largest pool of prosthetic mesh repairs of abdominal wall defects ever reported in Southeast Nigeria. The main limitation of this study is the fact that the design and follow up did not make provision for assessment of factors that predict recurrence, mortality and LOHS. Also, the design did not incorporate the non-mesh suture repair arm in order to compare outcome measures of mesh and non-mesh repairs. A more elaborate cross-sectional analytical study is recommended.

## Conclusion

The most common indication for reconstruction of complex abdominal wall defect in our environment is IH while financial impediment is the commonest barrier to early surgical treatment. The use of mesh to reconstruct these defects is largely safe and effective, but infection-related events are the major independent predictors of recurrence (mesh or wound infection) and mortality (systemic sepsis in diabetics). The implication lies with the possibility of rising unmet need for large abdominal wall defects in our setting due to increasing rates of laparotomy including caesarean section (that commonly initiate IH) and dwindling economic fortune (most common barrier to repair). Recommendation: There is urgent need to expand the coverage of postgraduate surgical skill acquisition training in our medical industry in order to cover doctors working in general and private hospitals; this will reduce the current spate of incisional hernias that commonly trail caesarean sections, gynaecologic procedures and other forms of failed abdominal wound closure. Greater awareness on danger of obesity for the general population and surgical patients in particular is salutary. There is also need to increase the coverage of National Health Insurance Scheme to cover biomaterials like mesh implants for hernias.

### What is known about this topic

- Long standing, neglected and voluminous midline defects and hernias are common in Africa;
- The uptake of prosthetic implants for abdominal wall defects is low in Nigeria;
- The recurrence rate after suture-based repair of abdominal wall defect is higher than figures after tensionless mesh implantation.

### What this study adds

- Presents the largest regional series in Nigeria to combine abdominoplasty and mesh implantation;
- Presents the lowest recurrence rate from both mesh and non-mesh repairs of complex abdominal wall defects performed in the past in Southeast Nigeria;
- Present the first data from Ebonyi province on the use of prosthetic implants to reconstruct complex midline abdominal defects.
